# Extending the Shelf-Life of Meat and Dairy Products via PET-Modified Packaging Activated With the Antimicrobial Peptide MTP1

**DOI:** 10.3389/fmicb.2019.02963

**Published:** 2020-01-09

**Authors:** Marta Gogliettino, Marco Balestrieri, Rosa Luisa Ambrosio, Aniello Anastasio, Giorgio Smaldone, Yolande T. R. Proroga, Rosalba Moretta, Ilaria Rea, Luca De Stefano, Bruna Agrillo, Gianna Palmieri

**Affiliations:** ^1^Institute of Biosciences and Bioresources, National Research Council (IBBR-CNR), Naples, Italy; ^2^Department of Veterinary Medicine and Animal Production, University of Naples Federico II, Naples, Italy; ^3^Department of Agricultural Science, University of Naples Federico II, Naples, Italy; ^4^Department of Food Microbiology, Istituto Zooprofilattico Sperimentale del Mezzogiorno, Portici, Italy; ^5^Institute for Microelectronics and Microsystems, National Research Council (IMM-CNR), Naples, Italy; ^6^Materias Srl, Naples, Italy

**Keywords:** antimicrobial packaging, antimicrobial peptide, shelf-life, spoilage microorganism, cytotoxicity

## Abstract

Fresh products are characterized by reduced shelf-life because they are an excellent growth medium for a lot of microorganisms. Therefore, the microbial spoilage causing significant food supply losses has become an enormous economic and ethical problem worldwide. The antimicrobial packaging is offering a viable solution to tackle this economic and safety issue by extending the shelf-life and improving the quality and safety of fresh products. The goal of this study was to investigate the effects of a food contact surface of polyethylene terephthalate (PET) functionalized with the previously characterized antimicrobial peptide mitochondrial-targeted peptide 1 (MTP1), in reducing the microbial population related to spoilage and in providing the shelf-life stability of different types of fresh foods such as ricotta cheese and buffalo meat. Modified polymers were characterized concerning the procedure of plasma-activation by water contact angle measurements and Fourier transform infrared spectroscopy measurements in attenuated total reflection mode (ATR-FTIR). Results showed that the MTP1-PETs provided a strong antimicrobial effect for spoilage microorganisms with no cytotoxicity on a human colon cancer cell line. Finally, the activated polymers revealed high storage stability and good reusability. This study provided valuable information to develop alternative antimicrobial packaging for enhancing and extending the microbial quality and safety of perishable foods during storage.

## Introduction

Short shelf-life of fresh foods represents one of the main limitations for the commercialization of this class of products, mainly due to their high content in nutrients and superficial moisture which leads to the fast growth of spoilage and pathogenic microorganisms (Aymerich et al., 2008; Patsias et al., 2008; Zhou et al., 2010). Indeed, it is well-known that microbial growth on the surface of a product is often responsible for the undesirable changes in flavor, aroma, and other organoleptic characteristics of fresh foods, which lower their quality and shorten their commercial life (Mead, 2004; Samelis, 2006; Petrou et al., 2012). Although the exact figure of the total economic loss due to food spoilage is hardly to estimate, it is clear that it constitutes an enormous financial burden (Blackburn, 2006) accounting for 1.3 billion tons per year by FAO (Cichello, 2015; Bondi et al., 2017). Therefore, even a reduction in food waste of 20–25% could save between $120 and $300 billion per year according to a recent report by the UK Waste & Resources Action Programme (WRAP). As a preservation technique, the refrigeration is necessary to maintain the microbial quality of fresh products, but it does not guarantee by itself a long shelf-life, which in the case of some foods amounts to a time period of about 4–5 days. Therefore, demand for safe fresh products presents major challenges to the food industry to develop innovative strategies for improving the preservation process and prolonging the storage time maintaining both the natural appearance and safety of foods by reducing or eliminating spoilage bacteria. A significant support in this field derives from the use of packages, which not only act as a barrier against moisture, water vapor, and gases, but they may also serve as a carrier of active substances in the “active packaging,” thus increasing the shelf-life and assuring the safety and/or quality of food products (Suppakul et al., 2003). Active packaging is the most relevant innovative idea applied for consumer satisfaction. It can be defined as a mode of packaging in which product, package, and the environment interact in a positive way to extend shelf-life of products and/or to enhance safety or sensory properties while maintaining the quality of the foods (Suppakul et al., 2003). Among the active packaging technologies, antimicrobial packaging is considered one of the most promising. These systems are based on the immobilization of antimicrobial agents on the surface of polymers, whose usage has strongly increased due to their large variety and the different compositions available, which make possible to adopt the most convenient packaging solutions, focusing on the specific needs of each product. One of the most common support that has found increasing applications within the packaging field is the polyethylene terephthalate (PET), a simple long-chain polymer, whose chemical inertness and physical properties have made it particularly suitable for different food applications. However, the chemical inertness of PET makes necessary to activate and functionalize its surface with specific treatments as the cold plasma before proceeding with the subsequent immobilization of bioactive compounds such as essential oils, plant extracts, bacteriocins, or enzymes (Jordá-Vilaplana et al., 2014; Malhotra et al., 2015). Some antimicrobial packages use immobilized antimicrobial peptides (AMP) to suppress the growth of microbes (Malhotra et al., 2015). AMPs are part of the innate immune system of all multicellular organisms (Andreu and Rivas, 1998; De Smet and Contreras, 2005; Guaní-Guerra et al., 2010) and include a chemically and structurally heterogeneous family, whose members have been isolated from a wide variety of animals, plants, bacteria, fungi, and viruses (Andreu and Rivas, 1998; Reddy et al., 2004). Nevertheless, three main characteristics that are shared by almost all known AMPs, can be distinguished: small size, highly cationic character, and tendency to adopt amphipathic structures (Nakatsuji and Gallo, 2012). These physicochemical properties make AMPs able to interact with the negatively charged microbial membranes. However, to serve as effective coating agents, the AMPs must meet several prerequisites, which include the retention of broad-spectrum antimicrobial activity once bound to packaging materials. As many naturally occurring peptides lack the ability to retain these properties, there is a need to develop new and more effective AMPs, with the aim to increase safety and shelf-life of food products. Recently, starting from the human source sequence of CPT-1a (McGarry and Brown, 1997), a new AMP, named mitochondrial-targeted peptide 1 (MTP1), was designed as already stated in Palmieri et al. (2016) and characterized. Specifically, the 15-mer peptide was revealed to be highly stable in a broad range of pH (2–10) and temperature (15–90°C) for prolonged incubation times. Moreover, MTP1 assumed α-helix/β-sheet structures in mimicked cell membrane solutions, as revealed by CD analyses. Finally, the compound exhibited significant bactericidal activity against *Listeria monocytogenes*, one of the most important foodborne pathogens.

The aim of the present study was to develop a new class of packaging materials, functionalized with the bactericidal peptide MTP1 and to evaluate both the usefulness and effectiveness of the aforementioned active coatings on the microbial quality and safety of fresh perishable products and the potential extension of their shelf-life.

## Materials and Methods

### Plasma Treatment

For plasma treatment and further peptide immobilization, the PET films were cut into disk-shaped pieces. Etching of PET disks was carried out using the PlasmaLab 80 Plus Reactive Ion Etching (RIE) system (Oxford Instruments, Abingdon, Oxfordshire, United Kingdom). The following parameters were modified to identify the best operative conditions: exposure time (*T*) (10–20–30–50–100–300 s); molecular oxygen concentration (O_2_) (10–50–100 sccm); partial gas pressure (*P*) (0.1–0.5 atm); and power of radiofrequency generator (RF) (50–100–300 W).

### Water Contact Angle Measurements

Water contact angles (WCA) were measured under static conditions by sessile drop method using an OCA 15EC system (DataPhysics Instruments GmbH, Filderstadt, Germany) coupled with a drop shape analysis software (SCA 20, DataPhysics Instruments GmbH, Filderstadt, Germany). A 1-μL drop was placed on the functionalized polymer surfaces, recording the images after 10 s. The WCA values are expressed as mean ± standard deviation (s.d.) of at least three measurements on the same sample in two independent experiments (i.e., at least six measurements for each result).

### Fourier Transform Infrared Spectroscopy

Fourier transform infrared spectroscopy measurements in attenuated total reflection mode (ATR-FTIR) were carried out in the 4000–650 cm^–1^ spectral range with a resolution of 4 cm^–1^, using a Thermo-Nicolet Continuum XL spectrometer (Thermo Scientific, United States). The FITR measurements were performed under inert (N_2_) atmosphere. Spectra have been automatically corrected from the background using Ominc software (Thermo Scientific, United States).

### MTP1 Immobilization Procedure

After oxygen plasma exposure, the pre-activated PET films were incubated into a MTP1 solution of 50 μM concentration prepared in sodium phosphate buffer (PB; 10 mM), pH = 7.0 for 24 h at 25°C. After incubation, the liquid solution of unbound peptide was removed manually and the functionalized PETs were extensively rinsed in water and DMSO in order to remove the traces of non-covalently bound peptide before performing all the surface characterizations. The PET containers used in all the analyses were kindly provided by the dairy “Mini Caseificio Costanzo s.r.l.” located in Lusciano (Caserta, Italy).

### Immobilization Yield Analysis of PET Polymers

Immobilization yield analysis of MTP1 on pre-activated PETs was performed by using a reverse-phase high-performance liquid chromatography (RP-HPLC) system (Shimadzu, Milan, Italy). Once immobilization was completed, the supernatant solutions were recovered after 24 h incubation and chromatographically analyzed to indirectly estimate the amount of the peptide attached to the polymeric surfaces. For these analyses, 200 μL of the samples was injected over a μBondapak C18 reverse-phase column (3.9 mm × 300 mm, Waters Corp., Milford, MA, United States) connected to an HPLC system, using a linear gradient of 0.1% TFA in acetonitrile from 5 to 95%. A reference solution was prepared with the initial peptide concentration used for the functionalization procedure under the same reaction conditions and run in parallel. Therefore, by knowing the added peptide (reference solution), the amount of peptide not bound to the polymers (expressed as a percentage) was determined by comparing the peak area. A calibration curve of the C18 column using different MTP1 concentrations was built. All measurements were performed in triplicate on three different preparations.

### Release Test

The quantity of MTP1 released from the pre-activated PET disks was assayed by RP-HPLC following the same procedure previously described Peptide-immobilized PET slides were immersed in pure water or mozzarella cheese brine (1 mL) for 24 h at 4°C and then the recovered solutions were analyzed by RP-HPLC. The solutions in contact with the functionalized polymers at time *t* = 0 were used as control samples and run in parallel. All measurements were performed in triplicate on three different preparations.

### Shelf-Life Testing on Dairy Products

A total of three random samples of buffalo ricotta cheese (200 g) were collected from a dairy factory. MTP1-PETs disks of 2.5 cm diameter (surface of 4.91 cm^2^) were placed on the base of two Petri dishes. Non-functionalized PETs were used as control. From each package, 30 g of ricotta cheese was weighted and laid aseptically on disks inside Petri dishes and storage at 4°C. Microbiological analyses were performed at *t*0 (beginning), *t*1 (4 days), and *t*2 (10 days) in contact with the MTP1-PETs. After incubation, 10 g of ricotta samples was added to 90 mL of buffered Peptone Water in sterile stomacher bag and homogenized for 3 min at 230 rpm using a peristaltic homogenizer (BagMixer^®^400 P, Interscience, Saint Nom, France). Further 10-fold dilutions of the homogenates were made. Aerobic Plate Count (APC) and yeasts and molds were enumerated by spread plating on PCA incubated at 30°C for 48–72 h (ISO 4833-2:2013) and DRBC Agar plates incubated at 25 ± 1°C for 5 days for the colony count (21527-1:2008), respectively. The functionalized disks were washed three times by a sanitizing solution (Pursept-A Xpress, Schülke & Mayr GmbH, Germany) and exposed 2 h to UV radiations, before reusing.

### Shelf-Life Testing on Buffalo Meat

Water Buffalo (n.5), slaughtered in an EU authorized slaughterhouse at 34 months and live weight of approximately 470 kg was chosen. The half-carcasses were cold-stored (0 ± 3°C) for 5 days and then the sirloin steak muscles (SSM) from both sides of the animal were removed. Subsequently, SSM were placed, for prolonged dry aging, in a forced ventilation patented cell named “Maturmeat” (ARREDO INOX S.r.l.) with an automatic extraction system set at a temperature of 0°C and at HR values ranging between 68 and 70% at microbiological lab of Department of Veterinary Medicine and Animal Productions (University of Naples Federico II). At 90 days of the aging period, three sirloin steaks (SS) were chosen. This aging time was selected due to the best palatability of the meat increasing tenderness, flavor, and/or juiciness. Aseptically 50 g from steaks was cut and placed on plastic disk functionalized with MTP1 (9 cm diameter, surface of 64 cm^2^) inside Petri dishes and stored at 4°C. Analytical determinations were performed at *t*0 (beginning) and *t*1 (4 days) after contact with the peptide. APC was detected according to following procedures: 10 g of each sample and 90 mL of sterilized Peptone Water were placed in sterile stomacher bag and homogenized for 3 min at 230 rpm using a peristaltic homogenizer (BagMixer^®^400 P, Interscience, Saint Nom, France). Afterward, 10-fold serial dilutions of each homogenate were prepared in Peptone Water, followed by streaking in duplicate for APC performed according to ISO 4833-2:2013 on Plate Count Agar incubated at 30°C for 48–72 h and for yeasts and molds performed according to ISO 21527-1:2008 on DRBC plates incubated at 25 ± 1°C for 5 days. The functionalized disks were washed three times by a sanitizing solution (Pursept-A Xpress, Schülke & Mayr GmbH, Germany) and exposed 2 h to UV radiations, before reusing.

#### Physicochemical Analyses

The pH of samples was measured using a digital pH-meter (Crison-Micro TT 2022, Crison Instruments, Barcelona, Spain). The a_*w*_ (activity water) (Aqualab 4 TE – Decagon Devices Inc., United States) was determined by oven drying for 24-h at 105°C (AOAC, 1990). The 2-thiobarbituric acid (TBA) test (AOAC, 2000) was used to measure the lipid oxidation for each sample.

#### Rheological Analysis

On buffalo meat samples was performed: (a) Texture profile analysis (TPA), a compression test for determining the textural properties of meat pieces (Ruiz de Huidobro et al., 2005) by measuring the compression force developed by the texturometer (Shimadzu EZ test); (b) Colorimetric measurement using a Konica Minolta CR300 colorimeter (Minolta, Osaka, Japan). CIE *L*^∗^(lightness), *a*^∗^(redness), and *b*^∗^(yellowness) values were recorded for each sample. For all rheological analyses, the steak cores were collected in parallel to the muscle fibers, using a hand-held steel cork borer. A cylindrical 10 mm-diameter probe of ebonite was used for the TPA tests.

#### Sensory Evaluation

The sensory attributes of buffalo meat and ricotta buffalo cheese were estimated by a panel of five panelists (Altieri et al., 2005), which evaluated the following parameters: color, odor, taste, chewiness, and general appearance. The samples treated with not-functionalized PETs were used as control. Panelists scored each sample with a point scale, ranging from 0 (attributes most disliked) to 5 (attributed most liked). After storage period, the meat was cooked at 80°C for 10 min (to simulate the mode of administration in restaurant) for taste and chewiness evaluation. Two pieces of each sample, buffalo meat and ricotta cheese, were served to panelists at each sample time.

### Cytotoxicity Assay

The cytotoxicity of the immobilized MTP1 was tested against HT-29 cells, a human colon cancer cell line used extensively to study the effects of different food products on human health (Martínez-Maqueda et al., 2015). HT-29 cells (kindly donated by Dr. Rosanna Palumbo CNR-IBB, Naples, Italy) were grown in Dulbecco’s Modified Eagle Medium (DMEM) supplemented with 10% (v/v) fetal bovine serum (FBS) and 10 mM L-glutamine. After reaching log phase, cells were transferred into 24-well plates (1 × 10^5^/mL) and incubated for 24 h at 37°C. Therefore, MTP1-functionalized PET disks (surface of 80 mm^2^) were added in each well and incubated for 24, 48, and 72 h at 37°C. Experiments were performed in quadruplicate. After incubation, the medium was removed and the remaining adherent cells were washed with PBS, fixated with 10% formaldehyde solution for 15 min at room temperature. Samples were subsequently washed with water and stained with 10% crystal violet solution for 30 min. Cell viability was quantified by eluting the dye from the stained cells with 10% acetic acid. Absorbance was measured spectrophotometrically at 595 nm (Multiskan FC; THERMO). Cells with the addition of not-functionalized PETs were set as negative control.

### Statistical Analysis

All experiments were performed at least five times. Data were analyzed using GraphPad Prism 5.0 (GraphPad Software Inc., San Diego, CA, United States). Statistical analysis of microbial counts was performed by using Student’s *t*-test for independent data Sensory evaluations, TBA, a_*w*_, and pH data were analyzed by Student’s *t*-test for independent data. *p* < 0.05 was considered to be statistically significant.

## Results and Discussion

### Activation and Functionalization of PET Polymer by Cold Plasma and MTP1

Packaging has a fundamental role in ensuring the safe delivery of goods throughout supply chains to the end consumer (Lindh et al., 2016). In this context, polymeric materials cover a large section of requirements in the field of the food industry and provide support surfaces for the immobilization of biologically active molecules. Specifically, PET is actually one of the most common polymers used in the food packaging, because of its physicochemical and mechanical properties and it is also relatively inexpensive to produce (Siracusa, 2012). However, surface properties of PET are usually inadequate in terms of wettability and adhesion properties, so it should be modified in order to improve its desired surface features and enhance its suitability, prior to any further processing, such as functionalization with biologically active molecules. In recent years, one of the most interesting procedures that have been employed to overcome these disadvantages is gaseous plasma treatment (Jordá-Vilaplana et al., 2014; Pankaj et al., 2014). Because of this procedure, the PET wettability is enhanced, and a more hydrophilic surface is created, rendering the polymer suitable for the preparation of new materials. Indeed, the versatility of PET is based on the development of metastable reactive groups (-COOH^∗^, -OH^∗^) (Pankaj et al., 2014), which allow the covalent surface derivatization with the available chemical groups of biomolecules.

**FIGURE 1:**
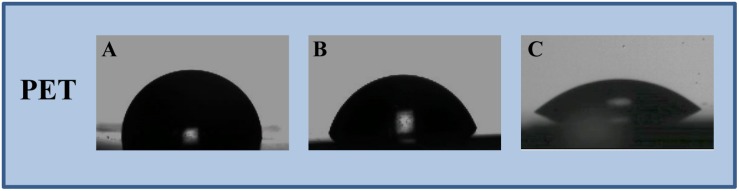
Water contact angle measurements performed on **(A)** pristine PET, **(B)** oxygen plasma activated-PET, and **(C)** MTP1-functionalized PET. The measurements were performed on five samples in duplicate.

**FIGURE 2:**
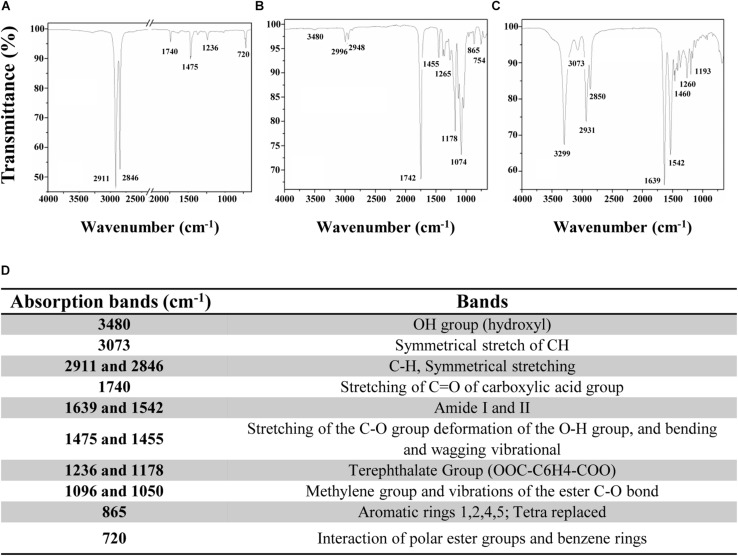
ATR-FTIR spectra of PET samples **(A)** before radiofrequency cold plasma treatment, **(B)** after plasma treatment, and **(C)** after MTP1 bio-conjugation. **(D)** The table reporting the main absorption peaks observed in ATR-FTIR spectra of PET samples.

In this study, an immobilization platform on PET surfaces by using the previously characterized AMP MTP1 (Palmieri et al., 2016) was developed, exposing PET disks to a radiofrequency cold plasma by changing the main parameters of the process including the O_2_ exposure time (*T*), RF power, and the concentration/the partial pressure (*P*) of molecular oxygen O_2_. To assess the surface hydrophilicity of the PET slides after treatments, water contact angle (WCA) measurements were performed (Figure 1). Prior to activation, WCA was found to be 89 ± 3° (Figure 1A), confirming the hydrophobic nature of PET surface, whereas after plasma treatment carried out at the optimal experimental conditions (50 W and 10 s of exposure time), the contact angle was reduced to 76 ± 2°, demonstrating that the plasma process significantly increased the hydrophilicity of the surface (Figure 1B). It is important to note that when higher values of RF power (RF = 300 W) and long exposure times (*T* = 100–300 s) were applied, a macroscopic change in the roughness of the PET surface was detectable, indicative of a material degradation process. Subsequently to the plasma procedure, PET samples were incubated for 24 h in MTP1 buffer solution. As shown in Figure 1C, the efficiency of the coupling reaction with MTP1 was confirmed by a strong increase in the surface hydrophilicity, as indicated by the pronounced decrease of WCA value (36 ± 3°). This phenomenon was due to the reaction of the peptide free chemical groups (typically -COOH and -NH_2_) with the reactive groups (-COOH^∗^, -OH^∗^) generated on PET surface by plasma activation (De Stefano et al., 2009). On the other hand, PET samples not pre-treated by radiofrequency cold plasma and incubated in same conditions in the presence of MTP1 showed the same value of WCA (75 ± 1°) of the pre-activated polymer, corresponding to negligible non-specific adsorption of the peptide on the PET surface. FTIR was also employed to confirm the success of bio-conjugation of MTP1 on PET disks. The ATR-FTIR spectrum of the not pre-activated PET (Figure 2A) displayed different main peaks corresponding to the C–C, C–H, and C–O bond stretching. After plasma treatment, the presence of the -OH group peaks in the FTIR spectra was detected (Figure 2B), consistently with the increase of the surface wettability quantified by WCA measurements (Socrates, 1994; De Stefano et al., 2013). The appearance of the characteristic absorption signals of the peptide, including the Amide I and Amide II bands, which arise from the peptide bonds that link the amino acids (O=C–NH) residues in the MTP1 sequence, confirmed the covalent bonding of the peptide on the PET surface (Figure 2C). Specifically, the Amide I band located between 1650–1560 cm^–1^ is produced mainly by the C=O stretching vibration of the peptide bond, while the absorption associated with the Amide II band at higher frequencies in the 1580–1490 cm^–1^ interval led primarily to bending vibrations of the N–H bond (Figure 2C). The main absorption peaks observed in ATR-FTIR spectra of PET samples are listed in the table reported in Figure 2D. Control samples not subjected to radiofrequency cold plasma treatment and incubated in aqueous peptide solution displayed an FTIR spectrum almost identical to that of Figures 2A,B (data not shown) (Edge et al., 1996; Silverstein and Webster, 1998; Chen et al., 2013).

### Coupling Yield and Stability of MTP1-Immobilized PET Disks

In order to consider PET materials as promising candidates for applications in the food industry as antimicrobial packaging that are able to efficiently increase the food quality and safety, the AMPs used as coating agents must retain several prerequisites, including the broad activity spectrum and the biocompatibility. Nevertheless, the immobilization of AMPs is still challenged by suboptimal coating strategies leading to (i) inadequate surface concentrations and (ii) loss of antimicrobial activities with non-specific binding chemistry culminating in changed orientations of the peptide molecules and/or associated cell toxicities. Therefore, it is firstly important to develop an effective surface tethering strategy that would impart the desired antimicrobial characteristics of the targeted biomaterial. This challenge strongly depends on the appropriate peptide concentration required to ensure a high efficiency of the immobilization procedure used and a great surface coating.

In this work, RP-HPLC analysis was employed to quantitatively measure the surface-immobilized yield starting from different MTP1 concentrations. Using this method, the amount of the PET-tethered peptide was indirectly evaluated by comparing the peak area (in the RP-HPLC-chromatograms) of the peptide not bound to the polymer after the bio-conjugation, with that of the initial peptide concentration used (at *t* = 0). The data obtained from these analyses demonstrated that the immobilization efficiency was concentration-dependent, reaching the highest yield (53%) using 50 μM, corresponding to a surface coverage of 11.2 nmol/cm^2^ of PET. The chromatographic profile obtained for the MTP1 50 μM concentration and used to calculate the immobilization yield is reported in Supplementary Figure 1. The coupling yield was further confirmed by interpolation using a six-point calibration curve, which was generated utilizing known MTP1 concentrations (measured using an analytical balance) (Supplementary Figure 1 *insert*).

An important pre-requisite for AMP-coated packaging is the stability of the immobilized peptides. Therefore, the functionalized PET disks were incubated under different physiological conditions (i.e., immersion in pure water or in a representative liquid food matrix such as mozzarella cheese brine) up to 24 h, and the peptide-release was analyzed by RP-HPLC. As shown in the chromatograms reported in Supplementary Figure 2, both pure water and mozzarella brine did not cause MTP1 leakage from the PET polymers after 24 h incubation either at 4 or 25°C. In addition, no peptide-release was detectable even after prolonged incubation times (until to 72 h) in all the conditions analyzed (data not shown), thus highlighting the high stability of our system, that makes it an appropriate candidate for food applications.

### Effect of MTP1-PETs on the Microbiological Quality of Dairy Products and Meat

Fresh food such as dairy products and meat because of their specific composition represent good support for a rapid growth of spoilage microorganisms that strongly influence the storage life of this class of products. In the case of cheese and meat, parameters such as water activity, pH, temperature, types, and viability of contaminating microorganisms are reported as some of the key factors that affect their rate of spoilage. Therefore, it is not surprising that these foods differ widely in spoilage characteristics. Among the troublesome microorganisms, yeasts and aerobic psychrotrophic Gram-negative bacteria can be considered the main causative agents of microbial spoilage and therefore they are recognized indicators of the hygienic quality of the foods. Indeed, psychrotrophic bacteria can produce large amounts of extracellular hydrolytic enzymes, and the recontamination of food products with these bacteria is determinant for their shelf-life, while yeasts are responsible of the main food degradations. These microorganisms are able to grow under a great variety of conditions and to survive in different environments resulting in unwanted physical and chemical changes, altering texture, smell, taste, or appearance of fresh products and rendering them not feasible for human consumption anymore. Therefore, extending the shelf-life of meats and dairy products represents the main challenge for food companies, and it is vital because, in the real world, these products do have fixed lifetimes after which they will perish.

**FIGURE 3:**
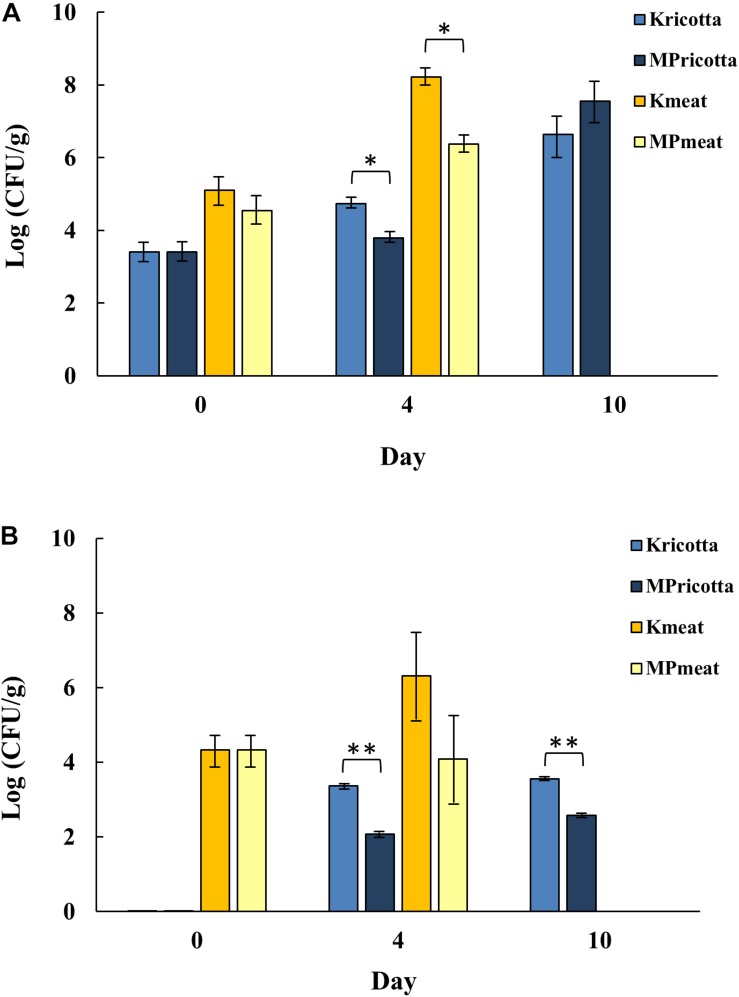
Evolution of **(A)** aerobic plate count (APC) and **(B)** yeast counts of ricotta cheese and meat, treated with MTP1-PETs (MP) during different days. Not-functionalized PETs were used as control (K). Data are presented as means ± s.d. of five different samples analyzed in triplicate. ^∗^, Significant difference (*p* < 0.05) between the treated and the control samples; ^∗∗^, Significant difference (*p* < 0.01) between the treated and the control samples.

In this context, the effectiveness of MTP1-PETs was evaluated through a comparison of the development of total aerobic mesophilic bacteria (APC) and yeasts on ricotta cheese and meat samples, stored under refrigeration (4°C) 1 day more than the normal shelf-life set by the company and analyzed in order to simulate a commercial storage time interval. With the aim to determine the possible influence of MTP1-PETs on the microbial growth in the products under investigation, the evolution of APC and yeast counts of samples treated with not-functionalized PETs (control) was assessed (Figure 3). Firstly, the initial values of the microbial counts were approximately in the range usually found for this variety of foods (4–5 Log CFU/g). As expected, bacterial microorganisms were able to proliferate in the control samples, with APC values ranging from 4–8 Log CFU/g during the period of monitoring (Figure 3A). On day 4 of treatment, meat samples in contact with MTP1-PETs were characterized by a significant (*p* < 0.05) bacteria growth inhibition (2.0 ± 0.2 Log CFU/g) respect to those exposed to the control PETs. It is worth to note that the microbiological acceptability limit of 7 Log CFU/g, as defined by the International Comission on Microbiological Specifications for Food (ICMSF) (1986), was reached in the control samples in the 4 days of storage, while this microbial count was observed in the treated samples after 6 days of storage with a concomitant worsening of the sensorial characteristics of the meat. These results indicated that MTP1-PET films might be an effective coating to extend the fresh meat shelf-life, which is generally estimated to be 2–3 days beyond a sell-by date.

Concerning ricotta cheese samples, as reported in Figure 3A, a statistically significant 1 Log reduction (*p* < 0.05) of APC in the MTP1-PET samples was observed respect to control after 4 days of storage. Besides, no significant differences in APC values were detected between samples of ricotta cheese stored on the PETs activated or not at day 10 of treatment, probably indicating the limited effect of long storage time on ricotta cheese preservation of the functionalized polymers (Figure 3A).

Similar results were obtained by evaluating yeasts growth (Figure 3B). After 4 days of storage 1 Log CFU/g increasing was revealed in meat products on control PETs in contrast to values of yeast counts equivalent to those determined at *t*0 and measured for MTP1-PET meat samples. As far as ricotta cheese is concerned, both at 4 and 10 days of treatment, it was observed the same significant (*p* < 0.01) trend of yeasts count reduction (1 Log CFU/g) confirming the possible effectiveness of the active PET to prolong the shelf-life of this kind of products. Interestingly, the results obtained for ricotta cheese samples are very notable considering that the yeasts constitute the most representative microbiological typology that strongly affects the shelf-life of this class of products, although no limits were fixed by European rules. However, Argentinian food regulations set up a maximum limit at no >3.5 Log CFU/g yeasts for cheeses with water content >55%, such as ricotta. As shown in Figure 3B, PET-coated ricotta samples did exceeded this limit until the end of storage (10 days) while the control samples reached the limit already after 4th day.

**FIGURE 4:**
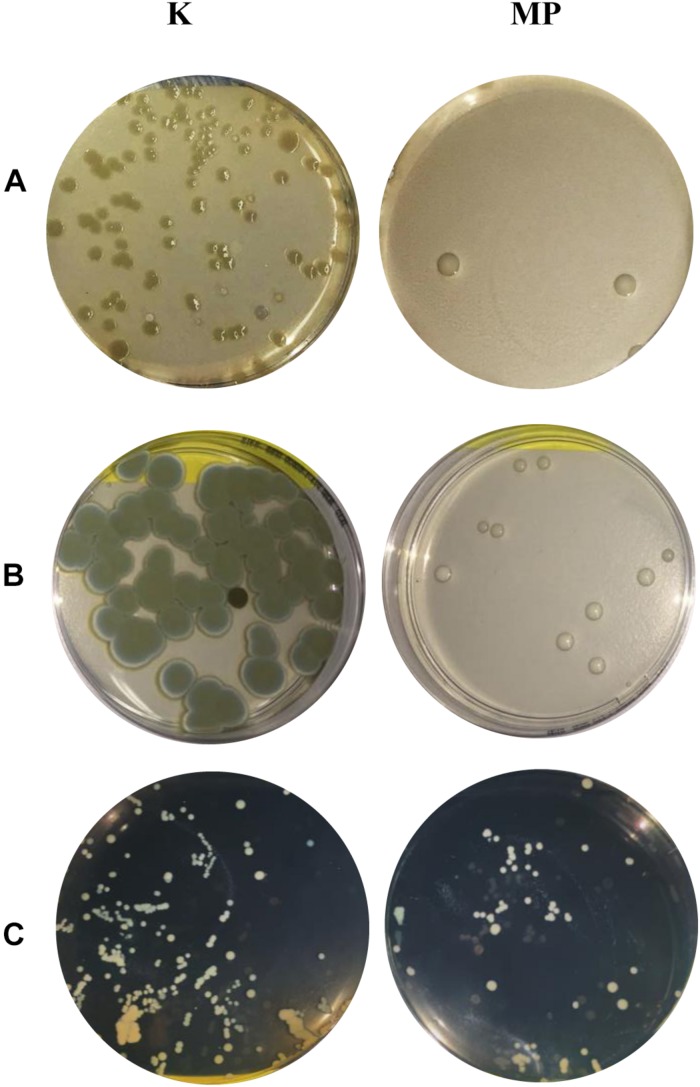
Representative **(A)** APC and **(B)** yeast plates of meat, and **(C)** APC plates of ricotta cheese incubated in the presence of not-functionalized PETs (K) or MTP1-PETs (MP) during 4 days.

Remarkably, the same results in terms of APC and yeast counts were determined even after three times of reuse of MTP1-PETs. Representative APC and yeast plates were reported in Figure 4.

### Effect of MTP1-PETs on Physicochemical, Rheological, and Sensory Quality of Dairy Products and Meat

During storage, food products undergo physicochemical, rheological, and sensory changes that may affect their organoleptic qualities and in turn, discourage their consumption. In this context, it is essential for a packaging to preserve also the inherent qualities of a product during storage beyond the microbiological safety. Therefore, a detailed analysis concerning the aforementioned parameters was performed on the buffalo meat and ricotta cheese samples treated or not with MTP1-PETs at the investigation times. All data are reported in Tables 1, 2.

**TABLE 1:** Effects of MTP1-PETs on physical–chemical (pH, a_w_, and TBA test), rheological parameters (color and texture), and sensory evaluation of buffalo meat samples.

	**Day 0**	**Day 4**				
		**Control**		**MTP1-PETs**		
		***M***	**SD**	***M***	**SD**	***t*-test**
pH	5.630	5.700	−	5.810	−	−3.024^∗^
a_w_	0.983	0.978	−	0.980	−	–2.450
TBA test	0.673	0.768	−	0.722	−	–5.489^∗∗^
Lightness	28.270	32.643	10.570	31.143	16.910	0.701
Redness	10.753	7.700	2.460	10.863	2.390	3.398^∗^
Yellowness	5.583	3.180	9.880	5.913	3.320	–1.843
Chroma	16.337	10.880	18.460	16.777	8.450	−2.785^∗^
Hue angle	0.479	0.392	0.090	0.499	0.010	–0.976
Adhesiveness	–35.643	–29.420	−	–22.187	0.080	–6.320^∗∗^
Cohesion	39.178	31.583	1.470	32.345	0.130	0.080
Hardness	343.142	336.928	2.320	331.237	2.010	0.223
Cohesiveness	0.384	0.365	0.020	0.357	0.010	–0.452
Friability	121.750	128.428	1.900	127.654	0.980	–0.541
Elasticity	0.781	0.784	0.010	0.779	0.060	–0.014
Gumminess	137.002	126.784	1.410	121.350	2.010	0.418
Chewiness	104.887	99.949	1.620	101.279	6.820	–0.385
Resilience	0.215	0.196	0.010	0.192	0.020	–0.260
Color	4.200	2.800	0.800	3.400	1.200	–189.737
Taste	4.400	3.600	1.200	3.800	0.800	–0.632
Odor	3.600	2.800	0.800	3.200	0.800	–141.421
Chewiness	4.400	3.400	1.200	3.800	0.800	–126.491
Overall acceptance	4.400	2.800	0.800	3.800	0.800	−3.536^∗^

*Levels of significance of treatments: ^∗∗^*p* ≤ 0.01; ^∗^*p* ≤ 0.05. *M*, means; SD, standard deviations.*

**TABLE 2:** Effects of MTP1-PETs on physical–chemical (pH, a_w_, and TBA test) and sensory evaluation of buffalo ricotta cheese samples.

	**Day 0**	**Day 4**					**Day 10**				
		**Control**		**MTP1-PETs**			**Control**		**MTP1-PETs**		
		***M***	**SD**	***M***	**SD**	***t*-test**	***M***	**SD**	***M***	**SD**	***t*-test**
pH	6.770	6.900	0.010	6.860	−	0.894	6.950	−	6.950	−	–0.229
a_w_	0.983	0.989	−	0.991	−	–0.695	0.989	−	0.991	−	–118.695
TBA test	0.009	0.010	−	0.012	−	–0.133	0.015	−	0.012	−	1.270
Color	4.800	4.400	1.200	4.800	0.800	–126.491	3.000	2.000	3.600	1.200	–1.500
Taste	4.200	4.000	−	4.200	0.800	–1.000	2.600	1.200	3.000	2.000	–1.000
Odor	4.800	2.800	0.800	3.600	1.200	−2.529^∗^	1.800	0.800	2.600	1.200	−2.529^∗^
Chewiness	5.000	3.800	0.800	4.200	0.800	–141.421	2.200	0.800	3.200	0.800	−3.535^∗^
Overall acceptance	4.800	3.600	1.200	4.400	1.200	−2.309^∗^	2.400	1.200	3.400	1.200	−2.887^∗^

*Levels of significance of treatments: ^∗^*p* ≤ 0.05. *M*, means; SD, standard deviations.*

Interestingly, a significant increase (*p* < 0.05) in the overall acceptance parameter was measured in the MTP1-PETs respect to the controls at each investigation time both for buffalo meat and ricotta buffalo cheese. On the contrary, the findings showed that the meat products had little differences in terms of color, taste, odor, and chewiness along the storage, but they were not statistically meaningful (*p* > 0.05), However, panelists indicated that the odor intensity in the MTP1-PETs ricotta cheese sample was more desirable than the control (*p* < 0.05) after 4 and 10 days of storage.

As far as the physicochemical parameters is concerned, there were no substantial changes in the pH and a_*w*_ values and in general no significant differences were found across all groups of meat and the dairy product following the MTP1-PET treatment during storage (Tables 1, 2). In addition, the levels of oxidative deterioration (TBA) did not change in any meaningful way between the MTP1-PETs samples and the controls but for buffalo meat samples after 4 days of contact (*p* < 0.01), suggesting that MTP1 had a slight effect on the lipid oxidation of meat products.

Color of foods is another important parameter to evaluate their quality, since the consumers associate it with freshness. As reported in Table 1, the not treated samples suffered a gradual lean browning during the retail period, reflecting in terms of an increase of *L*^∗^ and a decrease in *a*^∗^ and *C*^∗^ values respect to *t*0. Conversely, lower *L*^∗^ and greater redness (resulting in higher *a*^∗^ and *C*^∗^, *p* < 0.05) were found in the MTP1-PET meats compared with the controls at 4 days. Finally, the rheological analysis evidenced no significant change in the textural qualities of the buffalo meat samples following the MTP1-PET treatments.

### Cytotoxicity Testing of Immobilized MTP1

**FIGURE 5:**
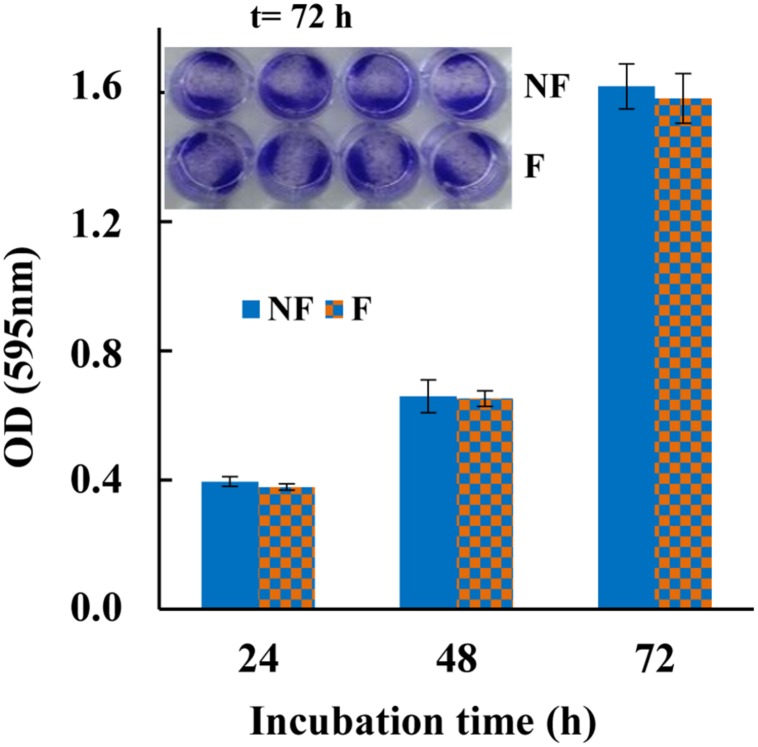
Cytotoxicity of MTP1-PETs on HT-29 cell line measured with crystal violet at 24, 48, and 72 h incubation. The data are shown as means ± s.d. of two separate experiments performed in quadruplicate. Insert: HT-29 cell line after staining with crystal violet incubated with MTP1-PET (F) for 72 h. Not-functionalized disks were used as control (NF).

In addition to antibacterial activity, cytotoxicity is another important parameter influencing the application of any material for industrial and medical purposes. Therefore, in order to ascertain the non-toxic behavior of MTP1-modified PETs, cell viability assay was carried out on the human colon cancer cell line HT-29, that is receiving special interest in studies focused on the effects of food products on human health (Martínez-Maqueda et al., 2015). To this aim, cell viability was determined by exposing these cells to functionalized polymers for different time intervals, using the non-conjugated PET disks as control. As reported in Figure 5, the mammalian cells remained viable up to 72 h of incubation with the functionalized PET, with no significant difference in cell viability in terms of absorbance between the controls and the MTP1-PET disks. These results indicated that the amount of peptide immobilized on PETs was not toxic to mammalian cells, thus suggesting that the projected polymers could be considered safe to be applied as antimicrobial packages in the food industry.

## Conclusion

In the field of food technology, innovative packaging represents an emerging solution that can confer many preservation profits on many food products. This study revealed that a 15-mer AMP could be covalently bound to the surface of PET materials, to produce highly stable antimicrobial packaging, which could be successfully used to improve the quality and safety of fresh products, maintaining the nutritional values during storage. The results obtained demonstrated the potential applicability of the MTP1-PET materials as not cytotoxic and safe active antimicrobial packaging through inhibition of the growth of spoilage microorganisms in relevant food model systems, such as the easily perishable ricotta cheese and fresh meat. The significant effects against bacteria in meat samples and yeast in ricotta cheese suggest a possible increase over the expiration date compared to the samples in contact with the control PETs. Although no significant variation was observed against yeast in meat samples and bacteria in ricotta cheese, we considered notable to underline the constant bacteria/yeast decrease in all samples stored on the PETs activated. Thus, the presented technology holds a great prospective for the development of highly effective bio-active and appealing packaging for food, preserving the microbiology quality and improving the safety of fresh products, with a concomitant extension of their shelf-life without adversely affect the organoleptic properties. However, long-term stability studies will be necessary in order to assure that the developed MTP1-PETs maintain stable performances over longer storage periods, which constitute an essential requirement for an innovative food packaging.

This work represents a pilot study, which provides a starting point to evaluate further the real potential application of MTP1-PETs as antimicrobial packaging in the food market.

## Data Availability Statement

All datasets generated for this study are included in the article/Supplementary Material.

## Ethics Statement

All experimental designs and animal care protocols were approved by the Animal Ethics Committee of the Buffalo Research Institute, Chinese Academy of Agricultural Sciences (BRI-CAAS) and Huazhong Agricultural University.

## Author Contributions

GP, LD, AA, YP, MG, and MB conceived and designed the experiments. BA, RA, GS, and RM performed the experiments. IR, MB, LD, and YP generated and analyzed the data. MG, GP, and AA wrote the manuscript. All authors contributed to the manuscript revision, read, and approved its submitted version.

## Conflict of Interest

BA was employed by Materias Srl. GP was employed by the government National Research Council (IBBR-CNR) and she was associated with the company Materias Srl. The remaining authors declare that the research was conducted in the absence of any commercial or financial relationships that could be construed as a potential conflict of interest.
