# Supplementary material for: Extending the Shelf-Life of Meat and Dairy Products via PET-Modified Packaging Activated With the Antimicrobial Peptide MTP1
Source: Front Microbiol. 2020 Jan 9;10:2963. doi: 10.3389/fmicb.2019.02963 (PMC6964532; doi:10.3389/fmicb.2019.02963)
Supplement: Supplementary file 1 [file Data_Sheet_1.PDF]

# *Supplementary Material*

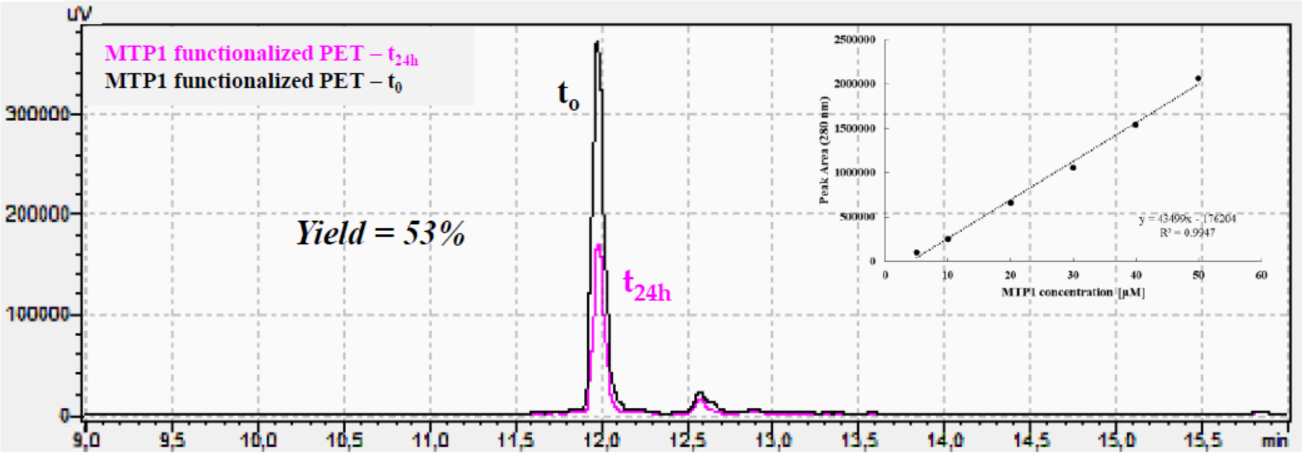


**Supplementary Figure 1.** Immobilization yield (%) of MTP1 on PET surface determined by reverse-phase HPLC chromatography on a C18 column after the coupling reaction (24 h). Pre-activated PET surfaces by plasma were incubated for 24 h with MTP1 (50 µM) in PB pH 7.0. The solutions recovered after incubation were further analysed. The peptide solution placed in contact with the pre-activated surface at time 0 (t = 0) was used as control. The chromatograms are representative of three independent experiments. ***Insert***: Calibration curve of the C18 column obtained using different MTP1 concentrations.

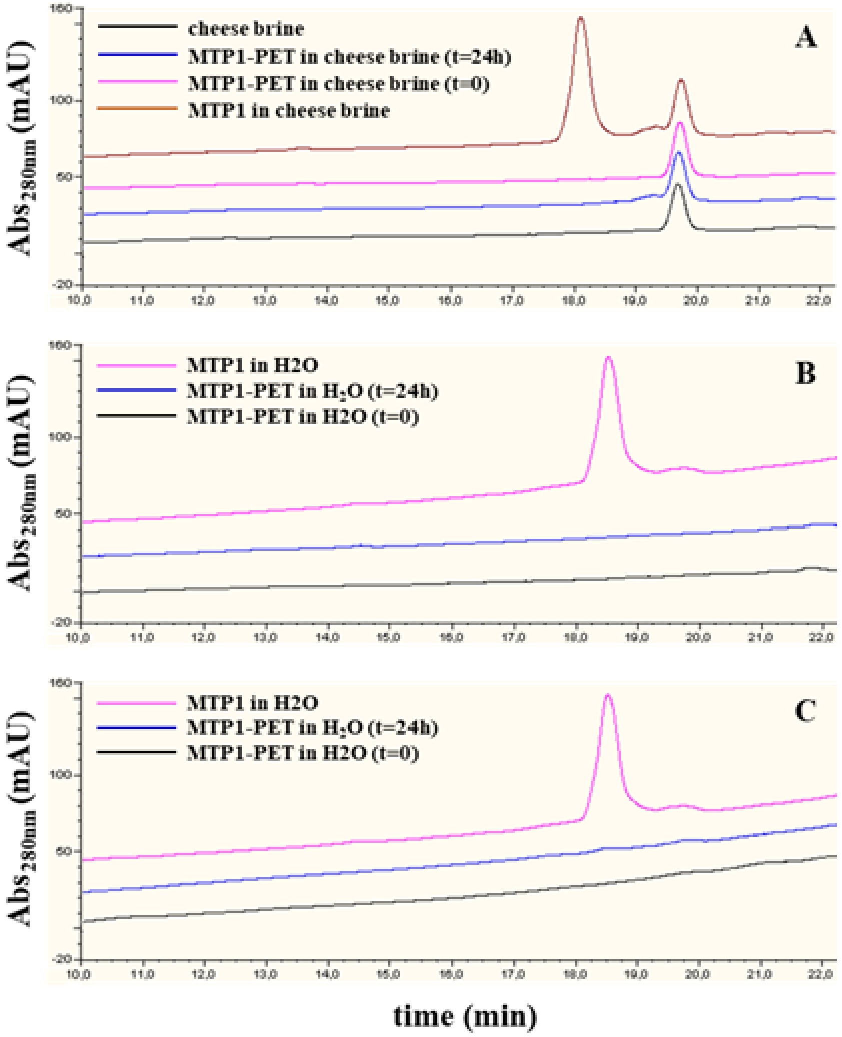


**Supplementary Figure 2.** Release analysis of MTP1 from functionalized PET performed by reverse-phase HPLC chromatography on a C18 column. MTP1-PETs were incubated for 24 in (**A**) mozzarella cheese brine at 4 °C or in pure water at (**B**) 4 °C or (**C**) 25 °C. After incubation, the solutions were recovered and injected on C18. The solutions in contact with MTP1-PET at time 0 (t=0) and MTP1 peptide (50µM) were used as controls.
